# Bullying of LGBTQ+ children and adolescents in schools: understanding the phenomenon, consequences, and international standards with a focus on the polish context

**DOI:** 10.3389/fpsyt.2024.1493745

**Published:** 2024-10-25

**Authors:** Bartłomiej Stańczykiewicz, Adrianna Senczyszyn

**Affiliations:** ^1^ Division of Consultation Psychiatry and Neuroscience, Department of Psychiatry, Wroclaw Medical University, Wroclaw, Poland; ^2^ Department of Psychiatry, Wroclaw Medical University, Wroclaw, Poland

**Keywords:** bullying, LGBTQ+, children, adolescents, mental health

## Abstract

Bullying refers to repetitive, aggressive behavior intended to harm or intimidate others. Cyberbullying extends this aggression to digital platforms, involving harassment via social media, texts, or emails. These forms of bullying are particularly damaging to LGBTQ+ youth, who often face discrimination based on their sexual orientation or gender identity. In the context of LGBTQ+ individuals, bullying and cyberbullying can lead to severe emotional and psychological harm, contributing to higher rates of depression, anxiety, and suicidal ideation. Despite extensive global efforts and campaigns to combat homophobia, LGBTQ+ students continue to face significant challenges, with the situation in Poland being notably severe. The review highlights various forms of bullying, including physical, verbal, and social aggression, and underscores the alarming involvement of school personnel in perpetuating such behaviors. The focus on the Polish context enriches the global discourse on LGBTQ+ rights and highlights the critical need for targeted interventions to support vulnerable youth in regions with prevalent conservative and religious sentiments.

## 1 Introduction

Clark, Kitzinger, and Potter (1), in the context of homophobic bullying, which is a problem for young non-heteronormative individuals, used the phrase “kids are just cruel anyway” in the title of their article. A significant example of this title is one of many suicides of a non-heterosexual teenager, 14-year-old Kacper, from Gorczyn, Poland. Kacper informed his family about being a victim of bullying, and they attempted to help him by transferring him to another school. Unfortunately, in the new school, he again faced bullying due to his sexual orientation, which most likely led him to commit suicide. Situations like this prompt reflection on how to effectively support LGBTQ (Lesbian, Gay, Bisexual, Transgender, Queer) individuals and protect them from community violence.

Bullying is described in the literature as repetitive and systematic harassment, teasing, or molestation of another person and can have both short- and long-term consequences. Bullying takes many forms, including verbal, physical, or psychological. The reason a person becomes a victim of aggressive and deliberate bullying can be, for example, religion, wealth, clothing, physical traits, disability, or sexual orientation (2, 3). Cyberbullying is bullying with the use of digital technologies. It may involve actions such as harassment (including insults or threats), spreading false information, impersonating someone, revealing private information, or deceit (gaining someone’s trust only to share their secrets online), as well as social exclusion (preventing someone from participating in activities). These behaviors can take place through email, instant messaging, text messages, social media platforms like Facebook or Tumblr, and other online channels (4, 5). Most victims of cyberbullying are also victims of “traditional” bullying (6). Recent international research on the phenomenon of cyberbullying conducted in eight European countries (Bulgaria, Cyprus, France, Greece, Spain, Hungary, Italy, and Poland) with 4847 participants aged 7-19 years shows that the highest level of peer violence is in Bulgaria and Hungary, and the lowest in Spain (7).

Social and cultural factors play a significant role in the prevalence and nature of bullying. Cultural norms, religious beliefs, and regional attitudes toward LGBTQ individuals can either exacerbate or mitigate the occurrence of bullying. In regions with strong conservative or religious sentiments, LGBT individuals may face more intense bullying compared to areas with more liberal and accepting views (8). Polish research on the phenomena of bullying and cyberbullying involving 1052 students showed that primary school students are more often victims of bullying, while perpetrators and victims of aggressive behavior are more often junior high school students. Moreover, the phenomenon of cyberbullying increases with age, which may be related to less parental control and greater access to the Internet (9). Selekman and Vessey (3) noted that many adults today remember when they were either perpetrators, victims, or witnesses of such behavior. Chronic bullying can have immediate health-related consequences, such as increased stress, anxiety, depression, headaches, and sleep disturbances (10). However, it is only prospective research from the last decade that has revealed that bullying in childhood or adolescence is causally related to the development of mental health problems in later years, including depression, anxiety, and suicidal tendencies that persist into adulthood (11).

This publication aims to examine the phenomenon of bullying concerning LGBTQ children and adolescents in school, understand its short- and long-term consequences, and present international standards for combating bullying in the school environment. The search was conducted using databases such as PubMed, PsycINFO, and Google Scholar. Keywords included “LGBTQ youth,” “bullying,” “cyberbullying,” “mental health,” “adolescence,” and “school interventions.” Inclusion criteria focused on studies published between 2000 and 2022, peer-reviewed journals, and research related to LGBTQ youth in educational settings.

## 2 Bullying and cyberbullying against LGBTQ individuals in school

The stigmatization of the LGBTQ community permits society to diminish and delegitimize these individuals, fostering prejudice and heterosexism—a belief that heterosexuality is the only natural, normal, and acceptable sexual orientation. This happens despite media coverage of LGBT rights issues (12). Unfortunately, LGBTQ individuals already experience the first signs of persecution in school. A meta-analysis conducted by Friedman et al. (13) showed that sexual minority individuals are 17 times more likely to be victims of bullying by their peers at school than non-minority individuals and 24 times more likely to skip school due to fear. Furthermore, it was found that differences between these groups in terms of peer bullying remained unchanged between 1990 and 2000.

Bullying in the context of LGBTQ individuals can take various forms. Research conducted by D’Augelli (14) showed that 81% of young LGB individuals experienced verbal insults, physical violence, intimidation, physical assaults, assaults with a weapon, and sexual assaults (respectively 38%, 15%, 6%, 16%). The comprehensive research conducted by the organization GLSEN (the Gay, Lesbian & Straight Education Network) found that as many as 84.9% of LGBT students often heard the word “gay” in a negative context (e.g., “that’s so gay”) at school, 71.3% encountered homophobic comments such as “fag,” and 61.4% faced statements related to not being masculine or feminine enough (15). Moreover, it is highly unprofessional and alarming that a significant proportion of LGBTQ individuals (56.9%) encounter such behavior from teachers and other school personnel (15). Besides such epithets, pushing, and beatings, there is also a form of social aggression by excluding LGBT individuals from peer groups (16).

Recent studies in Poland highlight the severity of bullying against LGBTQ+ youth. According to the “Rainbow Europe Index” published by ILGA-Europe, Poland ranks as the worst country in the European Union for LGBT rights (17). The report “Situation of LGBTQ+ Youth in Poland” by the Campaign Against Homophobia (KPH) indicates that 70% of LGBTQ+ students in Poland have experienced verbal violence, and 44% have experienced physical violence due to their sexual orientation or gender identity (18). Furthermore, nearly 30% of LGBT students in Poland felt unsafe at school due to their sexual orientation or gender identity, and 50% had experienced cyberbullying. These figures are significantly higher than those reported in many other European countries, indicating a particularly hostile environment for LGBT students in Poland (18). Moreover, Polish schools lack sexual education that sensitizes children and youth to issues of diversity. Polish research on the situation of young LGBT individuals from 2016 showed that only 15% of parents accept their child’s non-heterosexual orientation from the beginning, and there is a lack of systemic support for LGBT youth in schools (19).

The dominant understanding of LGBTQ students’ school experiences has been shaped by discourses that reduce “the problem” to bullies who express homophobic attitudes by targeting LGBTQ peers. In consequence, interventions mainly target eliminating harassment behaviors and providing protection for victims. Within this framework, cultural privileging of gender normativity, which is the expectation to conform to traditional gender roles based on one’s birth sex, as well as heterosexuality, goes unquestioned, LGBTQ marginalization is reproduced and re-rooted in new ways, and schools avoid responsibility for complicity in LGBTQ aggression. Predominant discourses of bullying have gained so considerable power in educational contexts as well as in public awareness that it has become practically impossible to understand school violence outside the “binary logic of protecting” (i.e., “victims” of bullying) and denigrating (i.e., pathologizing the “bully”) (20). This dominant narrative assumes schools are neutral sites where all students have an equal opportunity to succeed, ignoring institutional heteronormativity and heterosexism that shapes interactions within school spaces (20). Therefore, the majority of anti-bullying interventions function in the aforementioned binary opposition and do not include the cultural roots of aggression, as well as the role played by schools in the systemic marginalization of LGBTQ students.

## 3 Emotional and physical consequences of bullying in LGBTQ individuals

“Bullying in our school mainly happens in verbal form, but it hurts just as much as physical pain…” – this is one of the statements of an LGBTQ student, which indicates that individuals who experience bullying or persecution due to their sexual orientation or gender expression are particularly vulnerable to consequences such as reduced mental well-being (15). In the short term, LGBT youth are more prone to substance abuse, intimidation, isolation, rejection, anxiety, and depression compared to the general population (21). Research conducted by Westefeld, Maples, Buford, and Taylor (22) showed that LGB individuals are significantly more likely to experience isolation, prejudice, loneliness, and suicide attempts, which, according to the authors, is not due to their sexual orientation itself, but may be related to homophobic persecution and lack of support. Transgender individuals, although less frequently studied, face similar, if not more severe, challenges. The article “Experiencing Violence and Enacting Resilience: The Case Story of a Transgender Youth” by Gloria T. DiFulvio (23) provides a qualitative case study of Katie, a transgender youth, to explore how experiences of victimization and resilience manifest in the lives of sexual minority youth. At the time of the interview, Katie was in eighth grade. Although her doctors and family believed she was too young for surgery or hormone treatment, she looked forward to turning 18 to undergo gender-affirming surgery. For years, Katie experienced violence and harassment from her peers, knowing she was different but unable to fully grasp why. Following her first suicide attempt, she began therapy and initially came out as a gay male at the age of 13. It wasn’t long before she realized she was transgender, a realization that marked a significant turning point, allowing her to accept her true self and develop a more positive self-image. For Katie, the messages she received about her sexual minority identity prevented her from fully understanding her emotions and coming out to others. These experiences caused her to feel isolated, humiliated, and ashamed of her gender expression, which affected her sense of self. Katie mentioned that for as long as she could remember, other kids had teased her because she was different:

“Basically, I came out because kids at school were being very violent and rude with words and my principal had to tell my mom, “You know, I’m worried, I’m concerned about his safety.”

Throughout her education, she avoided trouble and closer relationships with her peers because she felt she didn’t belong in the boys’ group, and the girls’ group didn’t favor her. In addition to facing violence from her peers, Katie discovered that the parents of other students also posed a threat to her safety. Her mother had to attend a meeting with the principal and a concerned parent who had contacted the school, demanding Katie’s removal. The influence of homophobia results in isolation, as individuals who befriend or spend time with sexual minority youth are often labeled as queer themselves. Being associated with a certain group can make someone a target for violence related to sexual orientation. In Katie’s case, the physical violence against her lessened because other students believed that even by touching her, they might be seen as “weird.” The experience of being seen as different and struggling to fit into a world that prioritizes heterosexuality and gender conformity leads to numerous negative outcomes. The constant attacks and humiliation Katie faced at school deeply affected her self-esteem, ultimately leading her to attempt suicide as a way to escape the pain of living as an outsider in a heteronormative society. She recounted the feelings that drove her to this act:

“The week before the MCAS [Massachusetts Comprehensive Assessment System]; it was Mother’s Day, and it was just not a good day. Me and my mom had been, you know, like regular teenager and mother, fighting, nagging at each other. So I was already mad and upset about everything, and things weren’t getting better. They were getting worse. And I came to the point in my life where nothing meant anything anymore. Things just didn’t go well, and you know, that morning I OD’d on Tylenol.”

Experiencing bullying and persecution in school can have long-term consequences in the form of mental health problems in early adulthood. Among them, in addition to the depression above, are anxiety disorders, personality disorders, and addictions (24). Often, post-traumatic stress disorders also appear, and LGB individuals, to cope with unpleasant memories of bullying, resort to alcohol or drugs, which can lead to addiction (25). Low self-esteem, dissatisfaction with life, or problems with social integration also result from difficult experiences (26). Significant problems that may affect the above conditions are also lower educational achievements in school by LGB individuals, often caused by negative attitudes toward school and more frequent truancy (27). This undoubtedly affects their self-esteem and, above all, might be a consequence of fear of unpleasant situations at school. The impact of bullying extends beyond mental health, affecting educational outcomes and prospects. Lower academic achievements and higher truancy rates among LGBT students can limit their career opportunities and socio-economic status later in life (27). Long-term consequences of bullying for LGBTQ individuals include enduring mental health issues, such as chronic depression, anxiety, and post-traumatic stress disorder (PTSD). These mental health problems can persist well into adulthood, often exacerbating other life challenges. For instance, individuals with a history of bullying may find it more difficult to maintain stable employment, form healthy relationships, and achieve educational goals. Studies indicate that the stress from bullying contributes significantly to the development of various psychiatric disorders (11). Moreover, chronic stress and social stigma associated with being LGBTQ can lead to increased susceptibility to physical health problems, including cardiovascular disease and immune disorders (28). Kardasz et al. (29) found that anxiety and attachment styles significantly influenced life satisfaction among the Polish LGBTQ+ community. The researchers also highlighted that the life satisfaction of the LGBTQ+ community was lower compared to that of cisgender heterosexuals. These health disparities highlight the need for comprehensive support systems and interventions that address both the immediate and long-term impacts of bullying on LGBTQ individuals (21).

In light of the above studies, it is clear that the frequency and prevalence of mental health disorders and psychological problems in the LGBTQ population is higher than in heterosexual people. In addition to the prevailing heteronormativity and prejudice of peers and teachers in the school environment, LGBTQ people are affected by minority group stressors, such as discrimination, rejection, internalized stigma, and anticipated prejudice, which contribute to heightened stress and adverse mental health outcomes. The concept elucidates the unique stressors experienced by individuals belonging to stigmatized social groups. Such stressors result from a hostile and stressful social environment that devalues their identity, leading to various adverse mental and physical health effects. Ilan H. Meyer’s Minority Stress Model (28, 30) explains the relationship between social stressors and health outcomes among minority populations, particularly LGBTQ individuals. According to the model, these individuals experience chronic stress due to social stigma, prejudice, and discrimination, which in turn contributes to negative health outcomes. Meyer emphasizes that the source of these mental health problems is living in a stigmatizing, prejudiced, and discriminatory environment. He identifies social stressors such as internalized homophobia, sheltered living, and the anticipation of rejection as prominent contributing factors. Furthermore, the Minority Stress Model underscores the significance of protective factors in alleviating the adverse impacts of minority-specific stressors on physical and mental health. Peer support, community connectedness, and family support are critical in mitigating the negative effects of these stressors, particularly among trans individuals (31). Peer support, as a form of social support from fellow minority group members, plays a vital role in buffering against the negative mental health outcomes, including suicidal ideation and behavior, associated with minority stress (31).

## 4 Aspects of prevention and anti-bullying interventions for LGBTQ individuals

The results of existing studies on the impact of bullying and persecution of LGBTQ individuals highlight the necessity of finding appropriate solutions to prevent the negative effects of these behaviors. Focusing on the school environment, family support, and peer support is crucial (32) Friedman et al. (13) stress the importance of implementing safety policies and training programs for staff who may interact with young individuals from sexual minorities, aiming to ensure their protection, support, and to prevent bullying. According to Hanlon (33), initiating dialogue with teachers about homophobia is essential. He underscores the need to confront personal fears to enable open discussions about homosexuality. Additionally, educating students through discussions on LGBT-related topics and issues is vital (33). Sexual minority individuals are sensitive to experiencing bullying and persecution, especially when they lack support from parents.

The results of such studies are a guideline for school psychologists to educate teachers, school administration, and, above all, parents, indicating the consequences that an unsupportive school and family environment can cause (14). Grossman and D’Augelli (34), discussing bullying against transgender individuals, also emphasize the importance of psychoeducation for parents and teachers and point to significant assistance in coping with the stress of living with such an identity. Effective intervention programs should include comprehensive training for school staff, inclusive policies, and supportive peer networks. Programs that have shown success in other regions, such as the national initiative designed to create inclusive educational environments, the Safe Schools Program in Australia, could serve as models for similar initiatives elsewhere (35). The program includes workshops, educational materials, and support for implementing inclusive policies. It offers resources and training for teachers, school staff, and students to foster respect and understanding of LGBTQ issues. Research indicates that schools participating in the program have reported a significant reduction in homophobic bullying and an increase in LGBTQ students’ sense of belonging (35). Additionally, international cooperation and the sharing of best practices can enhance the effectiveness of anti-bullying strategies globally.

LGBTQ support programs around the world have proven effective in a variety of contexts. For example, the Trevor Project in the United States offers crisis intervention and suicide prevention services specifically for LGBTQ youth. Evaluations of Trevor Project programs show significant decreases in suicidal thoughts and improvements in mental health among participants (36, 37). Similarly, Stonewall’s School & College Champion programs in the UK provide resources and training for teachers to create inclusive environments for LGBTQ students. Participating schools receive tailored support, access to educational materials, and guidance on implementing anti-bullying policies. Studies show that schools that have been involved in these programs report lower levels of homophobic bullying and higher levels of student well-being (37).

Research shows that schools implementing such supportive programs have observed long-term benefits. A study by Poteat et al. (8) demonstrated that LGBTQ youth attending schools with inclusive policies and programs exhibited lower levels of depression and anxiety over time and showed better overall mental health outcomes into young adulthood.

The European Union (EU) has adopted measures to tackle bullying of LGBTQ students through various directives and initiatives. The EU’s approach emphasizes the necessity of cross-sectoral collaboration, involving education, health, and social services to address bullying holistically (38). Schools are encouraged to adopt inclusive policies that celebrate diversity, provide training for staff on LGBTQ issues, and establish effective reporting and support mechanisms for victims of bullying.

Several countries have implemented national policies specifically aimed at protecting LGBTQ students from bullying. Finland, for example, includes LGBTQ issues in its renowned KiVa anti-bullying program. The program offers classroom activities, individual interventions, and online resources that address bullying based on sexual orientation and gender identity (39). The success of the KiVa program has led to its adoption and adaptation in various other countries.

In Australia, the Safe Schools Coalition Australia program was specifically designed to create safe and supportive school environments for LGBTQ students. This program provides resources and training for teachers to better support LGBTQ students and to incorporate LGBTQ-inclusive practices into the school curriculum (40).

In the United States, the Department of Education’s Office for Civil Rights has issued guidance on the rights of LGBTQ students under federal civil rights laws. The “Dear Colleague” letter clarifies that schools must protect LGBTQ students from bullying and harassment and that failure to do so may constitute a violation of Title IX, which prohibits sex discrimination in education (41). Schools are required to investigate incidents of bullying promptly and take appropriate action to ensure the safety and well-being of LGBTQ students.

International guidelines for supporting LGBTQ people emphasize the need for inclusive policies and practices. The United Nations’ Free & Equal Campaign advocates for the protection and promotion of the rights of LGBTQ people worldwide, offering recommendations for legal and social reforms (42). In addition, the World Health Organization (WHO) provides guidelines to address health disparities for LGBTQ people, highlighting the implementation of non-discriminatory healthcare practices (43). These guidelines serve as benchmarks for countries seeking to improve the well-being of LGBTQ populations by implementing supportive measures and tackling discrimination.

In Poland, the legal situation for young LGBTQ individuals is challenging, with limited protections against discrimination. Polish law does not explicitly prohibit discrimination based on sexual orientation or gender identity in schools, leaving LGBTQ students vulnerable. The Campaign Against Homophobia (KPH) reports that while some schools have adopted anti-bullying policies, the implementation is inconsistent, and many students still face significant challenges (44). Research indicates that comprehensive sexuality education, which includes discussions on LGBTQ issues, is often lacking in Polish schools, contributing to a climate of ignorance and prejudice (44). Despite these challenges, there are efforts to improve the situation. Advocacy groups are working to promote more inclusive policies and provide support for LGBTQ youth, aiming to create safer and more accepting school environments. These aspects are crucial. For example, Poland is a country where homophobic hate speech is a highly common category of derogatory language (45). Zochniak et al. (46) showed that exposure to homophobic hate speech reduces the well-being of LGBT+ people, especially among those who identify strongly with the LGBT+ community.

## 5 Conclusion

The research and analysis presented in this publication underscore the urgent need for comprehensive strategies to address bullying and cyberbullying against LGBTQ individuals in schools. The detrimental effects of such bullying on the mental and physical health of LGBTQ youth are well-documented, with studies highlighting significantly higher rates of depression, anxiety, substance abuse, and suicidal tendencies compared to their heterosexual peers. These issues are exacerbated by societal heteronormativity and heterosexism, which contribute to the marginalization and stigmatization of LGBTQ individuals.

Unfortunately, despite many anti-homophobia campaigns, teasing, pushing, and verbal abuse of LGBT individuals still occurs in the school environment. Additionally, the extremely concerning results of studies show the involvement of individuals who should protect all children and youth, namely teachers and school staff, in such behaviors. Therefore, it is very important not only to implement effective forms of intervention to prevent bullying but also to conduct further research that may contribute to reducing the suffering of LGBT individuals and their full acceptance by others.

Effective interventions must extend beyond merely addressing individual acts of bullying. They must also tackle the systemic and cultural factors that perpetuate discrimination and exclusion. Schools play a crucial role in this regard, as they are not neutral environments but are shaped by broader societal attitudes and norms. Therefore, anti-bullying programs need to include education about diversity, promote inclusivity, and challenge existing prejudices and stereotypes.

To protect LGBTQ youth effectively, the following measures are recommended:

1. Legal Reforms: Implement explicit anti-discrimination laws that protect LGBTQ individuals in the education system.
2. Inclusive Education: Incorporate comprehensive sexuality education that addresses LGBTQ issues and promotes diversity and inclusivity, especially in educational settings.
3. Support Systems: Develop and effectively fund support programs to provide mental health services, crisis intervention, and community support for LGBTQ youth.
4. Educator Training: Provide comprehensive training for teachers, school staff, and parents on LGBTQ issues, inclusive practices, and how to support LGBTQ students and kids effectively.
5. Community Engagement: Foster partnerships between schools, families, and LGBTQ advocacy groups to create a supportive network for LGBTQ students.

The recommended measures, such as implementing explicit anti-discrimination laws, incorporating inclusive education, and providing support systems, are derived from best practices identified in existing literature. These studies provide evidence of the positive impact of such interventions on reducing bullying and improving mental health outcomes for LGBTQ youth (15, 37, 47). By adopting these measures, it is possible to create safer, more inclusive environments that protect the rights and well-being of LGBTQ youth, ultimately reducing the prevalence and impact of bullying and cyberbullying in schools.
